# Editorial: Physiology and Cellular Mechanisms of Isothiocyanates and Other Glucosinolate Degradation Products in Plants

**DOI:** 10.3389/fpls.2015.01105

**Published:** 2015-12-09

**Authors:** Atle M. Bones, Masakazu Hara, John T. Rossiter, Ralph Kissen

**Affiliations:** ^1^Molecular Biology and Genomics Group, Department of Biology, Norwegian University of Science and TechnologyTrondheim, Norway; ^2^Faculty of Agriculture, Shizuoka UniversityShizuoka, Japan; ^3^Division of Cell and Molecular Biology, Imperial CollegeLondon, UK

**Keywords:** isothiocyanates, cellular functions, physiological effects, glucosinolates (GLS)

Isothiocyanates (ITCs), thiocyanates, and epithionitriles are produced enzymatically from sulfur-containing glucosinolates (GLS) by β-thioglucosidases (thioglucoside glucohydrolase EC 3.2.3.147) called myrosinases (Bones and Rossiter, 2006), whilst there is growing evidence that they have multiple roles in host plants. The role of ITCs in plant defense against pests and pathogens has been thoroughly described and multiple roles and functions for GLSs and their derived products suggested. These amongst others include a role as allelochemicals and in sulfur storage, water transport, heat tolerance, stomatal regulation, apoptosis, growth inhibition, and signaling.

There are many thousands of papers on the cellular effects of ITCs when used in microbial and animal/human cell studies. Sulforaphane is one of the most studied ITCs and in the Thompson ISI gives approximately 6000 hits, which highlights the importance of this natural product. Included in this topic is a large body of data on the chemopreventive effects of ITCs in cancer models and on their cytotoxicity against bacteria, fungi, nematodes and insects. In comparison there are considerably fewer studies of the physiological and cellular activities of ITCs in plant models (Hara et al., 2010, 2013). The aim of this specialist research topic was to highlight the physiological role of ITCs and other GLS degradation products in plants and plant cells as well as pointing toward future research.

Plants contain, or are able to synthesize on demand, a plethora of defense compounds, including GLSs, that provide a substantial and effective arsenal against pathogens and pests. A knowledge of the GLSs, their tissue and cellular localization and metabolism under various environmental conditions is important in the assessment of potential physiological effects. The response of four *Arabidopsis thaliana* accessions differing in GLS composition and formation of GLS degradation products were investigated by Witzel et al. (2015) following exposure to the hemi-biotrophic fungus *Verticillium longisporum*. The data showed that *V. longisporum* infections affected both GLS profiles and the composition of degradation products in accession- and organ-specific ways. The plant-pathogen interaction between *A. thaliana* and *Alternaria brassicicola* was studied by Calmes et al. (2015) and set out to explore the mechanism by which ITCs could promote cell death. A decline in oxygen consumption, accumulation of reactive oxygen species and depolarization of the mitochondrial membrane in *A. brassicicola* was observed following exposure to ITCs. Two potential major players in the *A. brassicicola* response to ITC were identified to be a MAP kinase (AbHog1) and a transcription factor (AbAP1).

The role of GLSs and their degradation products in the plant response to abiotic stress is poorly investigated. Based on comparative studies of wild type *A. thaliana*, the double mutant *myb28myb29* and the single mutant *myb28*, affected in transcription factors regulating aliphatic GLS biosynthesis, Martínez-Ballesta et al. (2015) provided results indicating a role for short-chain aliphatic GLSs in water retention under salt stress.

Plants produce compounds that can affect the growth and development of other plant species. Such allelopathic effects have been reported for GLS related metabolites and material from Brassicaceae species with a high concentration of GLSs. These plants can be used in the control of soil born pests or pathogens and also in weed control (see e.g., Aspects of Applied Biology 126, 2014). It is known that seed germination can be delayed and even prevented by ITC. Rivera-Vega et al. (2015) studied GLS breakdown products in processing water from the detoxification of *Boscia senegalensis* seeds (called hanza) prior to consumption. Wastewater from the treatment of seeds contained large amounts of methyl ITC, which inhibited seed germination of all species tested, suggesting a practical use of this waste water in weed control.

Broccoli is considered to be healthy food due to the content of GLSs and the benefits of the ITCs, namely sulforaphane, which are released when the fresh vegetable is crushed. The GLSs are degraded by the active myrosinase (depending on the cooking process) and/or by microbial degradation of GLSs in the gut. In their perspective Angelino et al. (2015) studied the generation of ITCs by gut microbiota when rats were fed broccoli for at least a week and how the release of these ITCs is affected by addition of excess thiols. In view of the health benefits of ITCs derived from microbial degradation of GLSs the authors recommend to stimulate the gut microbiome with 3–5 servings a week of brassica vegetables (Angelino et al., 2015).

Hydroxynitrile glucosides and GLSs are not usually found in the same species, but there are exceptions as in the case of garlic mustard (*Alliaria petiolata*). Feeding ^14^C-labeled methionine to excised leaves of *A. petiolata*, Frisch et al. (2015) showed that both alliarinoside (a hydroxynitrile glucoside) and sinigrin (a GLS) are synthesized from methionine. They further investigated the different steps of alliarinoside biosynthesis and suggest that the aglucon precursor of alliarinoside might be produced from sinigrin decomposition products, representing an unexpected pathway for the synthesis of hydroxynitrile glucosides. GLS hydrolysis products also seem to be at the origin of the hydrogen cyanide released by *A. petiolata*, pointing at the ability of *A. petiolata* to use both HCN and GLS degradation products in its defense against pests and diseases.

Aiming for exploitation of the cellular mechanisms and the cellular cytotoxicity of ITC, Øverby et al. (2015a) exposed rat bladder cancer cells (AY27) and *A. thaliana* leaves to different ITCs. They observed growth inhibition and disruption of microtubules indicating a universal cellular effect of ITC in animal and plant cells. Another study from the same group used allyl ITC to investigate the effect of ITC on the cell cycle in *A. thaliana* (Åsberg et al., 2015). The primary effect observed for Arabidopsis plants being that allyl ITC induced cells to enter and accumulate in the S-phase, which was suggested to be a part of the plant defense response. ITCs are highly reactive compounds and their presence at the cellular level are expected to be efficiently handled to avoid cellular damage to the host cells. Øverby et al. (2015b) showed that exposure of Arabidopsis plants to vapor allyl ITC depleted cellular glutathione and upregulated the expression of several glutathione *S*-transferases, possibly indicating their role in the detoxification/control of ITCs in plant tissue.

Very little is known about the functions of these GLS related compounds in the host plants and much more remains to be discovered. Nearly 25 years ago it was stated that “the large amount of substrates, myrosinases, and cleavage products reported may indicate that this system has more than one function in the plant” (Bones et al., 1991). Current knowledge clearly supports a multifunctional role. We hope that the present research topic will encourage and stimulate research aiming at resolving the complex roles and mechanisms of GLS degradation products in plant cells.

## Author contributions

Drafted the manuscript (AB, RK), contributed to writing and approved final version (RK, JR, MH, AB).

## Funding

Research was supported by the Norwegian Research Council through grants 214329 and 184146.

### Conflict of interest statement

The authors declare that the research was conducted in the absence of any commercial or financial relationships that could be construed as a potential conflict of interest.

## References

[B1] Angelino D. Dosz E. B. Sun J. Hoeflinger J. L. Van Tassell M. L. Chen P. (2015). Myrosinase-dependent and -independent formation and control of isothiocyanate products of GLS hydrolysis. Front. Plant Sci. 6:831 10.3389/fpls.2015.00831 PMC4593958 26500669

[B2] Åsberg S. E. Bones A. M. Øverby A. (2015). Allyl isothiocyanate affects the cell cycle of *Arabidopsis thaliana*. Front Plant Sci. 6:364. 10.3389/fpls.2015.00364 26042144 PMC4436579

[B3] Bones A. M. Rossiter J. T. (2006). The enzymic and chemically induced decomposition of GLSs. Phytochemistry 67, 1053–1067. 10.1016/j.phytochem.2006.02.024 16624350

[B4] Bones A. M. Thangstad O. P. Haugen O. Espevik T. (1991). Fate of myrosin cells. -Characterization of monoclonal antibodies against myrosinase. J. Exp. Bot. 42, 1541–1549. 10.1093/jxb/42.12.1541

[B5] Calmes B. N'Guyen G. Dumur J. Brisach C. A. Campion C. Iacomi B.. (2015). Glucosinolate-derived isothiocyanates impact mitochondrial function in fungal cells and elicit an oxidative stress response necessary for growth recovery. Front. Plant Sci. 6:414. 10.3389/fpls.2015.00414 26089832 PMC4452805

[B6] Frisch T. Motawia M. S. Olsen C. E. Agerbirk N. Møller B. L. Bjarnholt N. (2015). Diversified glucosinolate metabolism: biosynthesis of hydrogen cyanide and of the hydroxynitrile glucoside alliarinoside in relation to sinigrin metabolism in *Alliaria petiolata*. Front. Plant Sci. 6:926 10.3389/fpls.2015.00926 PMC4628127 26583022

[B7] Hara M. Harazaki A. Tabata K. (2013). Administration of isothiocyanates enhances heat tolerance in *Arabidopsis thaliana*. Plant Growth Regul. 69, 71–77. 10.1007/s10725-012-9748-5

[B8] Hara M. Yatsuzuka Y. Tabata K. Kuboi T. (2010). Exogenously applied isothiocyanates enhance glutathione S-transferase expression in Arabidopsis but act as herbicides at higher concentrations. J. Plant Physiol. 167, 643–649. 10.1016/j.jplph.2009.11.006 20031254

[B9] Martínez-Ballesta M. Moreno-Fernández D. A. Castejón D. Ochando C. Morandini P. A. Carvajal M. (2015). The impact of the absence of aliphatic glucosinolates on water transport under salt stress in *Arabidopsis thaliana*. Front. Plant Sci. 6:524. 10.3389/fpls.2015.00524 26236322 PMC4502342

[B10] Øverby A. Bævre M. S. Thangstad O. P. Bones A. M. (2015a). Disintegration of microtubules in *Arabidopsis thaliana* and bladder cancer cells by isothiocyanates. Front. Plant Sci. 6:6. 10.3389/fpls.2015.00006 25657654 PMC4303138

[B11] Øverby A. Stokland R. A. Åsberg S. E. Sporsheim B. Bones A. M. (2015b). Allyl isothiocyanate depletes glutathione and upregulates expression of glutathione S-transferases in *Arabidopsis thaliana*. Front. Plant Sci. 6:277. 10.3389/fpls.2015.00277 25954298 PMC4406002

[B12] Rivera-Vega L. J. Krosse S. de Graaf R. M. Garvi J. Garvi-Bode R. D. van Dam N. M. (2015). Allelopathic effects of glucosinolate breakdown products in Hanza [*Boscia senegalensis* (Pers.) Lam.] processing waste water. Front. Plant Sci. 6:532. 10.3389/fpls.2015.00532 26236325 PMC4500904

[B13] Witzel K. Hanschen F. S. Klopsch R. Ruppel S. Schreiner M. Grosch R. (2015). *Verticillium longisporum* infection induces organ-specific glucosinolate degradation in *Arabidopsis thaliana*. Front. Plant Sci. 6:508. 10.3389/fpls.2015.00508 26217360 PMC4498036

